# Myocardial infarction due to septic thromboembolism in chronic rheumatic heart disease

**DOI:** 10.4322/acr.2023.444

**Published:** 2023-08-28

**Authors:** Suneel Rachagiri, Aravind Sekar, Saurabh Mehrotra, Uma Nahar Saikia

**Affiliations:** 1 Post Graduate Institute of Medical Education and Research Centre, Department of Histopathology, Chandigarh, India; 2 Post Graduate Institute of Medical Education and Research Centre, Department of Cardiology, Chandigarh, India

**Keywords:** Aortic Valve, Embolism, Endocarditis

## Abstract

Chronic rheumatic heart disease (RHD) is the most troublesome complication of rheumatic fever. Extensive valvular scarring and ventricular remodeling due to pressure and volume overload occur in chronic RHD. Deformed valves are at potential risk for developing infective endocarditis (IE) with further systemic embolism. We hereby describe a case of a patient diagnosed with chronic rheumatic heart disease and severe ventricular dysfunction, planned for aortic valve replacement. The patient developed septic shock during a hospital stay. The autopsy revealed infective endocarditis in the aortic valve with septic thromboembolism in the peripheral branches of the coronary artery and early multifocal myocardial infarction changes.

## INTRODUCTION

Rheumatic Heart disease is the most common and the leading cause of infective endocarditis in developing countries.^1^ In the presence of bacteraemia deformed and scarred valves in RHD are at potential risk for the development of IE due to disrupted laminar blood flow across orifices.^2^ Further, the risk for systemic embolism is very high in IE.^3^ The brain, spleen, kidneys, liver, heart, and mesenteric arteries are potential embolization sites. The incidence of coronary embolism is low and ranges from 3% to 11%.^4,5^ Embolism is more frequent in the peripheral branches than in major coronary arteries. We hereby, describe a case of chronic rheumatic heart disease with early multifocal myocardial infarction changes due to septic thromboembolism from aortic valve vegetation, depicted at autopsy.

## CASE REPORT

A 47-year-old gentleman with a history of Rheumatic Heart Disease presented with complaints of shortness of breath (New York Heart Association -III/IV). He had undergone St Jude Medical valve (SJM) replacement 25 years ago for severe mitral valve rheumatic involvement. He also had a history of thrombotic occlusion of prosthetic valve twice in the past, for which thrombolysis was done. He was admitted twice last year with breathing difficulty symptoms (NYHA III/IV) and was found to have severe left ventricular systolic dysfunction, right ventricular dysfunction with moderate aortic stenosis and moderate to severe aortic regurgitation. He was managed conservatively with diuretics, digoxin, beta blocker, ACE inhibitor and oral anticoagulants. He was advised for aortic valve replacement. However, he was lost on follow-up.

On examination, the patient was conscious, afebrile, and anemic with no cervical or axillary lymphadenopathy. A midline sternotomy scar was present. Pulmonary examination revealed bilateral vesicular breath sounds with crepitations. Abdominal and neurological examinations were normal. Blood pressure was 85/61 mm Hg; pulse rate- 125/min, irregularly irregular; respiratory rate-22/min. Electrocardiogram showed right axis deviation, atrial flutter with variable block, left ventricular hypertrophy with strain pattern and ventricular ectopics.

2D-Echocardiogram showed a normally functioning prosthetic mitral valve with normal movement. There was no paravalvular leak or mitral regurgitation. The aortic valve was thickened, calcified, and showed severe aortic stenosis with Aortic valve area by Velocity time integral-0.5 cm2-. Severe aortic regurgitation (AR Pt1/2 188, AR Jet Height/Left ventricular outflow diameter=12/22) was also noted. Left ventricular ejection fraction-20-25%; Right ventricular systolic pressure-Right atrial pressure+51; Tricuspid Annular Plane Systolic Excursion (TAPSE)-17. Compression ultrasonography of both lower limbs showed no evidence of deep venous thrombosis. The laboratory findings at admission showed hemoglobin of 8.4 gm/dl (reference range [RR]; 13.8-17.2 gm/dL); total leukocyte count of 5300 cell/mm3 (RR; 4500-11000 cells/mm3); platelets of 79x103 cells/mm3 (RR;150-450x103 cells/mm3); serum creatinine-1.4 mg/dL (RR;0.8-1.2 mg/dL); serum albumin-3.2 mg/dL (RR; 3.4-5.4 mg/dL); AST/ALT-87/147U/L (RR; 7-55/8-48 U/L); Procalcitonin: 1.2 ng/mL (RR; 0.1-0.49 ng/mL). Blood and urine cultures were sterile at the time of admission. He was managed with diuretics, beta-blockers, and ACE inhibitors and planned for aortic valve replacement. Due to economic constraints, aortic valve replacement was deferred by relatives. He initially responded with adequate diuresis. He became febrile during his hospital stay after 33 days of admission. Later, he developed cardiorespiratory arrest, from which he could not be revived.

## AUTOPSY FINDINGS

**Figure 1:**
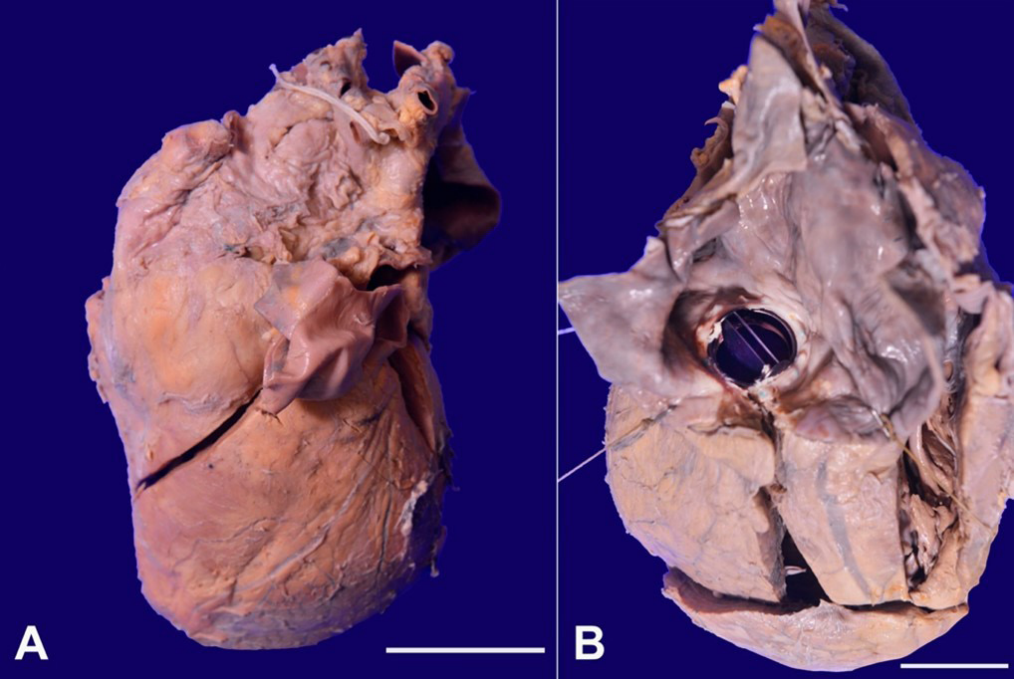
Gross view of: **A** – The heart is markedly enlarged and widely oblong weighing 700 g (scale bar= 4cm); **B** – Left atrium is markedly dilated with a hinged prosthetic valve with no thrombus or ring abscess formation (scale bar= 3cm).

A Partial autopsy was carried out with the deceased's relatives' full consent. The entire body was eviscerated via a midline thoracoabdominal incision. When the serous cavities were opened, the pericardial cavity yielded 40 ml of hemorrhagic fluid with blood clots. The heart was bulky and widely oblong, weighing 700 g (RR;330 g). The pericardial surface showed fibrinous tags (Figure 1A). The inflow/outflow technique was used to open the heart.

The left and right atrium was markedly dilated, and the endocardial surface showed wrinkling and sclerosis. The hinged prosthetic leaflet was in the mitral valvular region without any thrombus or ring abscess formation (Figure 1B).

**Figure 2:**
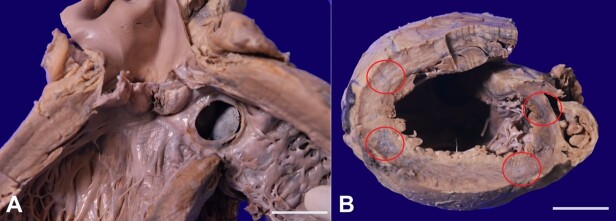
Gross view of: **A** – Aortic valves are markedly thickened with commissural fusion, and it shows residual vegetation in the ventricular surface of the left cusp and involving commissure (scale bar=2 cm); **B** – Cut surface of the heart after apical slice showing multifocal blackish discolouration involving anterolateral, inferior and posterior wall of the left ventricle (scale bar= 2 cm).

The tricuspid valve leaflets were thick and opaque. The right ventricle was hypertrophied with a wall thickness of 0.7 cm (reference range [RR] 0.4-0.6 cm). The root of the pulmonary artery and pulmonary valve annulus was markedly dilated. The left ventricular cavity was dilated, and the wall was hypertrophied with a thickness of 1.6 cm (RR 0.5-0.9 cm). The aortic valve cusps were markedly thickened and fibrotic with commissural fusion causing significant aortic stenosis. The ventricular surface of the left cusp and its commissural region with the posterior cusp was rough and showed residual vegetation measuring 0.5 cm at maximum (Figure 2 A). The cut surface of the heart shows multifocal blackish discolored areas involving anterolateral, inferior, and posterior wall of the left ventricle (Figure 2 B).

**Figure 3:**
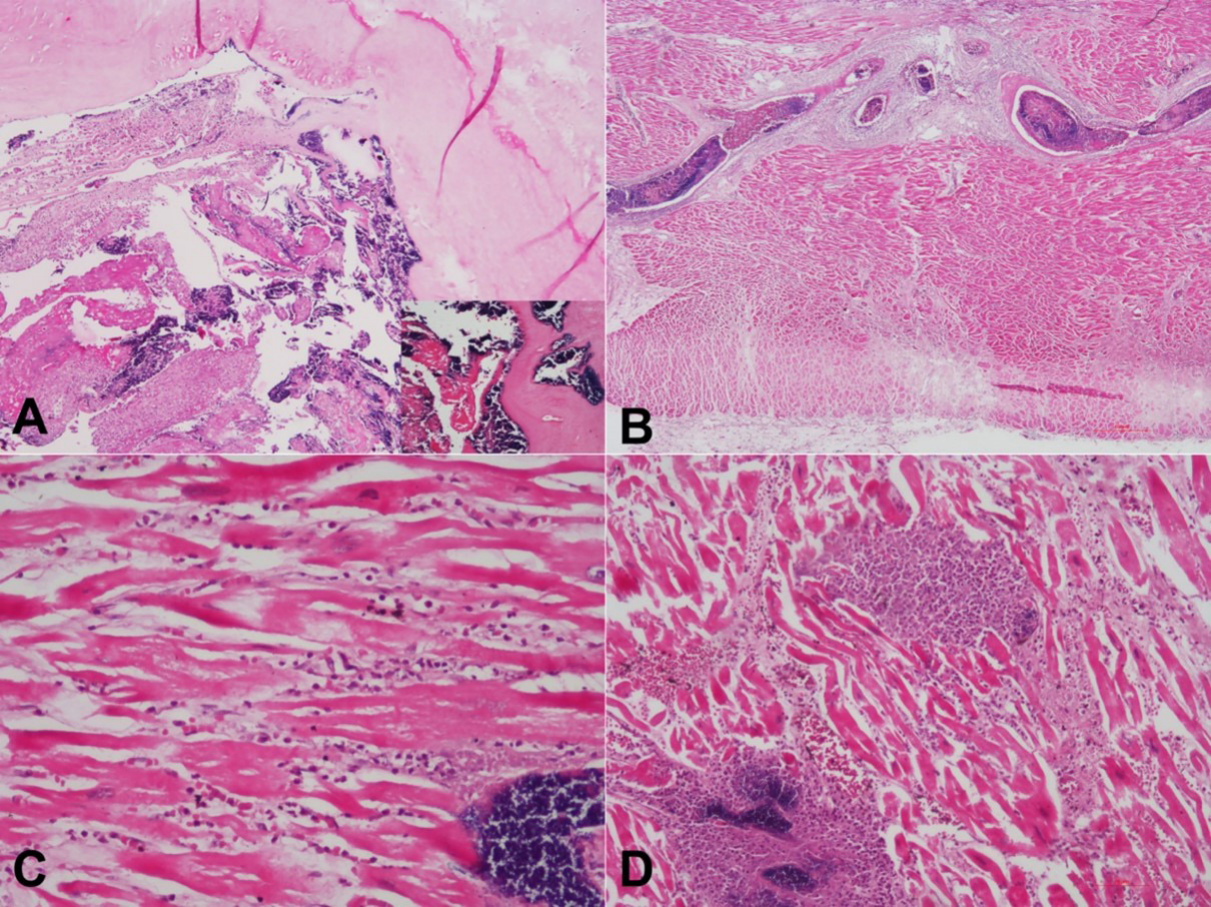
Photomicrographs of the heart. **A** – Microscopy of the aortic valve showing vegetation composed of fibrin, neutrophils-rich inflammation, and numerous bacterial colonies (Inset: Gram-positive cocci on gram stain) (H&E, 40X); **B** – One of the intramyocardial arteries showing Septic thrombo-emboli with near complete occlusion of lumina (H&E, 40X); **C** – Early infarct changes such as edema, neutrophil infiltration and extravasation of RBCs are seen in the surrounding myocardium (H&E, 200X); **D** – Micro abscesses around the intramyocardial vessels with seeding of bacterial colonies (H&E, 200X).

The histopathological examination of the aortic valve confirmed vegetation on its surface with numerous gram-positive bacterial colonies (cocci) accompanied by fibrin and neutrophil-rich inflammation (Figure 3A). Sections from the right and left ventricular walls showed septic emboli in many intramyocardial and pericardial arteries (Figure 3B). The surrounding myocardium showed early infarct changes of 18–24 hours duration, such as edema, neutrophils infiltration and extravasation of erythrocytes (Figure 3C). Myocardial abscesses with seeding of bacterial colonies were also seen around intramyocardial arterioles and capillaries (Figure 3D).

**Figure 4:**
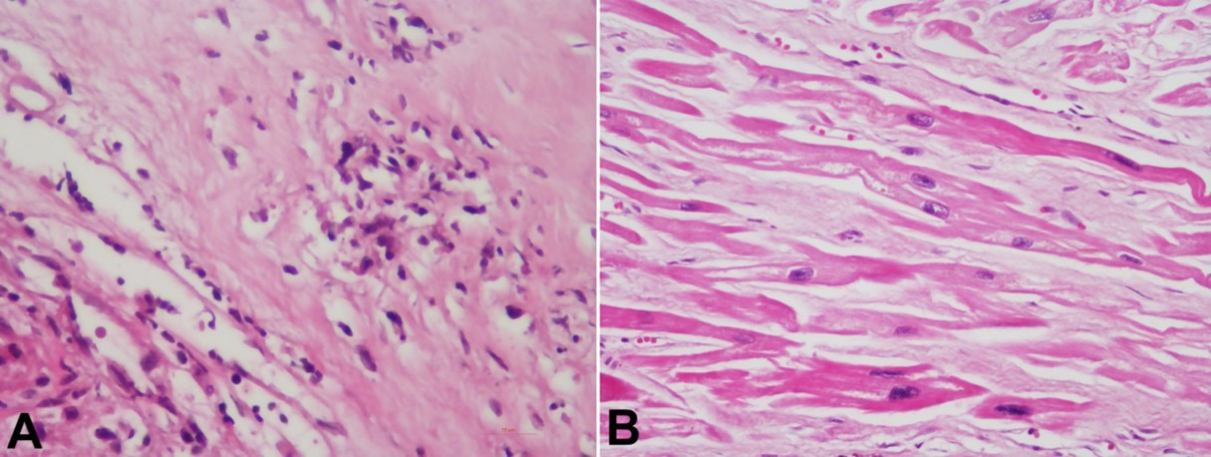
Photomicrograph of the: **A** – Aortic valve cusps are showing characteristic Aschoff’s nodules with caterpillar cells (H&E, 200X); **B** – Myocardium showing remodeling changes with enlarged myocytes containing boxcar-like nuclei and interstitial fibrosis (H&E, 200x).

The myocardial infarction was multifocal, involving diverse walls of the left ventricle, right ventricle, and interventricular septum, but it was not transmural. Coronaries were dissected and did not show any thrombus. Large areas of fibrosis with Aschoff's nodule and neovascularization were seen in aortic valve cusps (Figure 4A). In addition, marked remodeling changes due to pressure hypertrophy were noted in the form of hypertrophied myocytes with boxcar-like nuclei and interstitial fibrosis (Figure 4B).

Both lungs weighed 900 g (RR;1100-1500 g), and their cut surface was firm to feel with slight dusky discolouration. No pulmonary thrombus was seen. The microscopic examination of the lungs showed pulmonary arterial hypertension changes secondary to chronic passive venous congestion. The alveolar spaces showed marked edema and focal areas of hemorrhage and hemosiderin-laden macrophages. The pre-acinar and intra-acinar arteries were prominent, with concentric intimal thickening and medial hypertrophy. The pulmonary vein showed arterialization changes.

The liver weighed 1600 g (RR,968-1860 g) and presented a characteristic nutmeg appearance at the cut surface, with alternate dark and light areas. The microscopic examination of the liver showed maintained lobular architecture with marked sinusoidal dilatation and congestion, markedly in zone 3 with significant atrophy and necrosis of hepatocytes, indicating congestive hepatopathy. The spleen was markedly congested with a red pulp prominence and a white pulp depletion.

**Figure 5:**
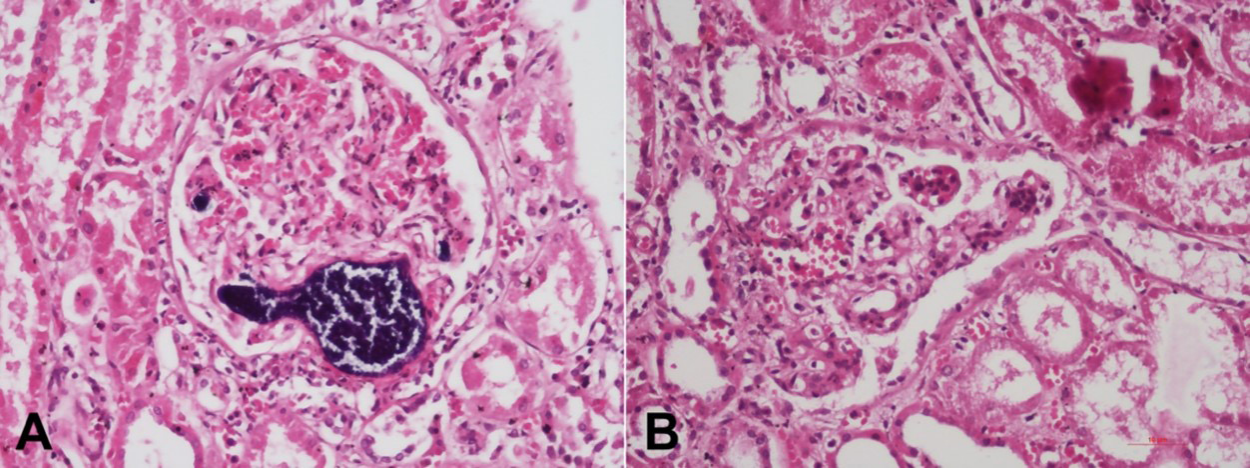
Photomicrograph of the kidney: **A** – shows septic thrombo-emboli clogging the glomerular capillaries and arterioles (H&E, 200x); **B** – Few glomeruli also depict segmental mesangial and endocapillary hypercellularity (H&E, 200x).

Both the kidneys weighed 300 g (RR,160-320 g) with cortical scars. The microscopic examination showed septic thrombo-emboli in glomerular capillaries, arcuate and interlobular arteries (Figure 5A). A focal and segmental endocapillary proliferative lesion (Figure 5B), with 1+ intense positive for Ig G on direct immunofluorescence was also noted. The stomach, small intestine, large intestine, adrenal, pancreas, skin, skeletal muscle, and bone marrow were normal.

## DISCUSSION

Chronic rheumatic heart disease is the most severe and worrisome complication of rheumatic fever. In India and tropical countries, the latent period for the development of a severe valvular dysfunction is shorter due to the higher risk of repeated infection and non-compliance with the treatment.^6^ Due to valvular scarring, dysfunction, and hemodynamic disturbances, pressure and/or volume overload occurs which results in extensive myocardial remodeling . Progressive cardiac hypertrophy, chamber dilation, and interstitial myocardial fibrosis may develop if valvar lesions are left untreated for an extended period.^7^ This can result in cardiac failure that may become irreversible even after valvar replacement or valvuloplasty.

Deformed valves are further prone to endothelial injury due to non-laminar blood flow and are at risk for infectious vegetation development in the setting of bacteremia. Vegetations more commonly occur in deformed mitral valves, followed by aortic valves. Prosthetic valves are also prone to vegetation. Further, vegetation may dislodge and cause septic thromboembolism.

Coronary embolisms in IE usually occur in the left anterior descending coronary artery and its branches because of its downward course compared with the right coronary artery or left circumflex artery.^8^ Due to its proximity to the coronary ostia, it is most frequently encountered in individuals with aortic valve endocarditis, as observed in the index case. Mobile vegetation, vegetation size >10mm, and infection with staphylococci or non-viridans streptococci are known risk factors for embolism.^9,10^ Furthermore, arrhythmic events in the RHD can potentially increase the risk of thromboembolism. The type of infarction depends on the size and location of the thromboembolism. If thromboembolism is significant, the primary coronary artery branch is obstructed, causing transmural myocardial infarction. If the embolism is modest, it travels to intramyocardial branches of coronary arteries that are smaller. As a result, multifocal infarction occurs and can involve interventricular septum and all the walls of ventricles. In addition, septic thromboembolism induces the spread of bacteria to the myocardium, which leads to micro abscesses and interstitial myocarditis.

The index case had a hospital stay of 33 days. Blood cultures were negative, and there was no evidence of vegetation on the 2D Echocardiography performed at the time of admission. Though the most sensitive transesophageal Echocardiography was not done at admission, endocarditis might have occurred later during the hospital stay. It is further supported by the abrupt onset of fever before demise. In the presence of extensive remodeling changes caused by Chronic rheumatic heart disease, multifocal myocardial infarction and interstitial myocarditis might further compromise ventricular function severely and cause death.

## CONCLUSION

Chronic RHD patients with a sudden development of fever must be evaluated for infectious endocarditis. Immediate transesophageal echocardiography must be conducted for early diagnosis to commence empirical antibiotic treatment and prevent septic embolization.
